# Assessing informed consent practices during normal vaginal delivery and immediate postpartum care in tertiary-level hospitals of Bangladesh

**DOI:** 10.18332/ejm/109311

**Published:** 2019-05-21

**Authors:** Md Abdul Karim, Syed Imran Ahmed, Jannatul Ferdous, Bushra Zarin Islam, Henok Ayalew Tegegne, Bachera Aktar

**Affiliations:** 1 BRAC, Dhaka, Bangladesh; 2 icddr,b, Dhaka, Bangladesh; 3 UNICEF, Dhaka, Bangladesh; 4 James P. Grant School of Public Health, BRAC University, Dhaka, Bangladesh; 5 European Food Safety Authority, Parma, Italy

**Keywords:** informed consent, normal vaginal delivery, respectful maternity care, tertiary-level hospital

## Abstract

**INTRODUCTION:**

This study was conducted to assess the informed consent practices during normal vaginal delivery (NVD) process and immediate postpartum care in the tertiary-level hospitals of Bangladesh.

**METHODS:**

A cross-sectional study was conducted at Dhaka Medical College Hospital (DMCH) and Sir Salimullah Medical College & Mitford Hospital (SSMCH) in November 2015. The study population and respondents were mothers who gave normal vaginal childbirth within the past 24 hours and received postpartum care in the study sites (N=190). The interview of every alternate mother from the patient register was conducted by researchers using a structured questionnaire. Descriptive analysis of findings was carried out using MS Excel 2013.

**RESULTS:**

The study findings revealed the complete absence of informed consent practices during NVD and postpartum care in the tertiary-level hospitals in Bangladesh. Consent (not informed consent) was taken from 95% of the mothers before proceeding with the NVD process, 50–72% of examinations (except breast examination, 0%) and 8–72% of procedures during postpartum care. Choice and preferences of mothers for taking an alternative process/examination/procedure were absent in all cases.

**CONCLUSIONS:**

The Respectful Maternity Care (RMC) Charter endorsed informed consent as one of the basic rights of child-bearing women. Absence of informed consent practices in the study sites indicates disrespect to maternity care and violation of this right. The Standard Clinical Management Protocols of Bangladesh also lacks clarification of this right. Improvement of the existing protocol, increased awareness and practices are essential to address protection of this right.

## INTRODUCTION

The term ‘informed consent’ has been in practice in the clinical setting since the late 1950s as part of patient and family rights^1^. Currently, its importance is such that it has been endorsed as one of the fundamental human rights of child-bearing women during the delivery process and postpartum care^1-3^. It is the process of communication between the health care providers and mothers regarding permission or refusal of specific interventions that plays a central role in promoting informed decision making between patients and practitioners to improve maternal and child health outcomes in the labour process^3,4^. Any examination, intervention or treatment especially for pregnant women without informed consent is considered an assault, and consent should be sought from the mother before proceeding examinations, delivery and postpartum care^4,5^. Studies conducted in health facilities worldwide indicate that women experienced some forms of disrespect or abuse, in the form of non-consented services during facility delivery and postpartum care, surgery, invasive procedure, anaesthesia, and use of blood products^6,7^. There are currently no available data on this issue in the context of Bangladesh, especially in tertiary-level hospitals.

The first aim of this study was to assess informed consent practices during normal vaginal delivery (NVD) process and immediate postpartum care in the tertiary-level hospitals of Bangladesh. The information from this study could inform practical measures and guidance to address this issue for further improvement of respectful maternity care.

## METHODS

A cross-sectional study was conducted in Dhaka Medical College Hospital (DMCH) and Sir Salimullah Medical College & Mitford Hospital (SSMCH) in Dhaka, Bangladesh in November 2015. The reason behind choosing this type of study was to quantify the prevalence rate of the inform consent practices in midwifery care in the study sites. The prevalence rate (9.7%) of informed consent practices at medical out-patient departments in tertiary-level hospitals in Pakistan was used for the calculation of sample size^8^. Based on that study’s findings and using the prevalence-based formula: n = t^2^p(1-p)/d^2^ where prevalence p = 9.7%, error margin d = 5% and confidence interval t = 98%, a sample size of 190 was calculated. The prevalence rate of Pakistan was used due to the very similar sociodemographic and cultural condition of the two countries. The respondents were mothers who gave normal vaginal childbirth within 24 hours, received postpartum care there, were in normal condition, whose newborns were alive, and were available in the study sites. Mothers who were not mentally and physically able to participate and/or in a risky condition, were excluded. A structured questionnaire was developed (Supplementary file) and pretesting was done in a similar setting (Shaheed Suhrawardy Medical College Hospital, Dhaka). Based on the experience during pre-testing, it was revised and made functional. Considering the previous patient flow record, the initial plan was to collect 117 (62%) and 73 (38%) samples from DMCH and SSMCH, respectively. However, based on the availability of the respondents during data collection, information of 49 (26%) and 141 (74%) mothers were collected from DMCH and SSMCH, respectively. The systematic random sampling method was used and every alternate mother from a hospital patient register was selected for an interview. If the selected mother fell under exclusion criteria, the next serial number was chosen. The face-to-face interview was conducted by the researchers using the questionnaire. Data were entered into a spreadsheet (MS excel 2013 version) and descriptive (numeric, graphical, and tabular) analysis of the findings was carried out.

In this study:

Normal Vaginal Delivery refers to — the spontaneous vaginal delivery of baby in the normal manner^9^.

Informed consent is defined as — verbal or written permission of mother/family member/relative (after being informed and understood the purpose, benefit, success rate and potential risk of examinations or procedures explained by the service providers) to service providers before proceeding delivery and any examination and/or procedure^5^. Consent refers to — only permission (written or verbal) of mother/family member/relative to the service provider to proceed delivery and any examination and/or procedure (without considering the explanation of the process/examination/procedure)^10^. Immediate postpartum care is defined as — maternal care within 24 hours of normal vaginal delivery.

Ethical approval of the study was obtained from the ethical review committee (ERC) of the James P. Grant School of Public Health (JPGSPH), BRAC University, Bangladesh, while written permission was obtained from the hospital authority for the study, and voluntary participation, consent and anonymity of participants were ensured. Nothing was done during the study that could interrupt the ongoing services in the study sites, and the regular activities of both mothers and service providers.

## RESULTS

### Profile of respondents

The mean age of the mothers was 23.71 years, the majority (58%) were below 25 years of age, and 38% of them came to the hospital for their 2nd delivery. More than half (52%) of the mothers completed only primary level education, and 96% were involved in household activities, and nearly half of them (43%) lived in an urban area (Table 1).

**Table 1:** Sociodemographic characteristics of the respondents*

*Variables*	*Frequency (%) N=190*
**Data collection site**	
DMCH	49 (26)
SSMCH	141 (74)
**Mother’s age (years)**	
<18	04 (02)
18–24	106 (56)
25–29	50 (26)
30–34	24 (13)
35–39	06 (03)
**Mother’s employment status**	
Employed	07 (04)
Household work	183 (96)
**Level of education**	
No Education	02 (01)
Signature only	23 (12)
Primary education	99 (52)
Secondary and above	66 (35)
**Living place**	
Rural	41 (22)
Urban	82 (43)
Peri-urban	67 (35)
**Current delivery status**	
1st child delivery	55 (29)
2nd child delivery	72 (38)
≥3rd child delivery	63 (33)

* Mothers who gave normal vaginal childbirth within the last 24 hours in SSMCH & DMCH and participated in the study.

### Informed consent practices

The findings show the complete absence of informed consent practices during the NVD process and immediate postpartum care in the tertiary-level hospitals of Bangladesh. Written consent (not informed consent) is taken to proceed with NVD, which is mostly signed by family members (92%), relatives (5%) but rarely by the mother (3%). This consent includes only a signature (by mother, family member, or relative) on the admission form used as a proxy for a consent form that contains the patient’s information such as name, age, date and purpose of admission. The purpose, benefits, success rate and potential risks of the procedure were not mentioned in the message above the signature. However, the purpose was explained verbally.

**Figure 1:**
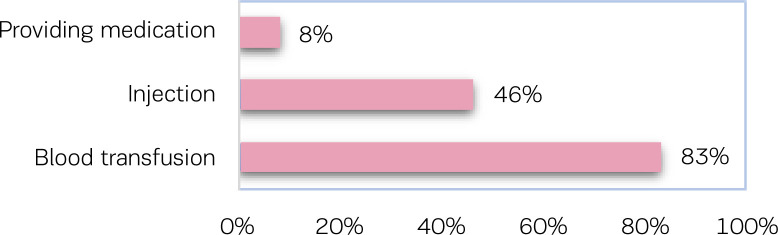
Consent taken for procedures during immediate postpartum care

During immediate postpartum care, verbal consent taking ranged from 50% to 72% of cases for examination, except (0%) for breast examination (Figure 1).

Verbal consent is also taken for invasive services, such as injection (46%) and blood transfusion (83%), and for the provision of medication (8%). Of the mothers who require the same examination/procedure more than once, only 12% were asked for verbal consent every time the examination/procedure proceeded. Only 11% of the service providers allowed mothers to ask questions if they wanted to have information about any examination and procedure.

## DISCUSSION

Informed consent is one of the components of respectful maternity care during the delivery process and the postpartum period^3^. It is not only a voluntary consent or refusal of any process or procedure; it is a universal right of child-bearing women that ensures their right to know in detail and be well-informed about the services (before agreeing to any care) they receive^1-4^. This right prevents the service providers from doing anything to child-bearing women without their knowledge and consent^3,4^. Any process or procedure especially for pregnant women without informed consent creates a communication gap between mothers and service providers regarding permission or refusal of certain interventions, hampers mother’s informed decision making, leads to non-consented care, disrespect and abuse in maternity care^1,4,5^. In many countries, this right is absent or poorly present, which indicates disrespect, abuse and violation of this basic human right of women in maternity care^3,8,10-14^. Non-consented care, disrespect, abusive behaviour and assault to the mothers are becoming emerging and urgent issues in different countries during maternity care^3,6^.

Findings from this study reveal the complete absence of the basic right of informed consent in the tertiary-level hospitals of Bangladesh, which indicates disrespect and the violation of this basic right of child-bearing women during midwifery care, NVD process, and immediate postpartum care^3,5^. The admission form of the child-bearing women is being used as a proxy consent form. Before proceeding NVD, the service providers obtain a signature on the admission form under the message: *‘I, at this moment giving the consent for NVD with my full sense and the hospital authority will not be responsible if any risks or bad things happen’.* They consider it as consent for the NVD process. This message does not explain the types of risks from the procedure. The potential risks are also not explained to the patient, family members or relatives by the service providers before the procedure. The child-bearing women, family members, or relatives only know that they have given a signature for the delivery process. However, earlier studies in different countries revealed poor adherence to obtaining the patient informed consent by the health professionals of medical out-patient departments and for proceeding with any procedure during childbirth in hospitals^5,8,14^.

Verbal consent is only the oral permission of mothers for proceeding with any type of examination required during immediate postpartum care. Mothers do not have any choice for any alternatives for the examinations or the opportunity to ask the service provider about the services they receive. They were destined to receive the services only in the way the service providers were ready to provide. Similarly, studies in different countries also revealed that mothers experienced these types of disrespect for examinations/procedures during the antenatal visit, delivery process and postpartum care in the hospitals^10,11^.

A study revealed the practice of obtaining verbal consent from mothers during procedures in postpartum care, whereas informed consent is in place for surgery, invasive procedure, anaesthesia, and using blood products^7^. Some other studies in different countries identified the non-compliance of the national standard protocols, raised ethical and legal questions, and suggested that the issue regarding respectful maternity care needs to be addressed^13,14^.

Although in other countries, one-third of the mothers had the opportunity to ask questions to service providers regarding any examination and procedure^15,16^, the present study found very few (11%) service providers allowed mothers to ask questions.

Many guidelines and protocols related to informed consent and the right of refusal of services have been established for several decades to ensure women’s rights to dignity and respectful care, in order to have facility-based childbirth with reduced risk of maternal mortality^4,6^. Still many countries have not addressed or clarified these rights in their standard operating protocols for clinical management^3,12^. Thus, disrespectful care is becoming an emerging problem that is creating growing concern in communities, from the wealthiest to the most impoverished nations worldwide^3,6^. This situation is more common in the health systems of low-income settings^6^. This type of non-consented care occurs because of the absence of clarification on this right in the standard operating protocols of clinical management, lack of understanding, low level of education, language difficulties, cultural background, and distressed condition of mother by the pain during the labour process^5,13,17^.

The Respectful Maternity Care (RMC) Charter endorsed informed consent as one of the universal rights of child-bearing women^3^. Consent taking is also mandatory for maternity care in Bangladesh^17^. However, this type of disrespect (non-consented care) occurred due to the absence of clarification on respectful maternity care in the standard operating protocols, the lack of knowledge by service providers on the issue, that lack of care incurs a penalty is not mentioned in the protocols, the condition of mother, and busy units in the tertiary-level hospital settings in Bangladesh^5,17^.

### Limitations

The results are based on the response of the mothers from only two tertiary-level hospitals. There are also very limited studies in the peer-reviewed literature on the issue. So, our findings could neither be compared with other studies in similar settings in Bangladesh nor worldwide.

Further studies are needed for the identification of the gap between RMC charter endorsed rights and the present standard protocols for clinical management in Bangladesh and their practice in reality. Development of a specific consent form and the orientation of service providers to protect the rights of child-bearing women are essential so that the practice of informed consent is implemented to ensure respectful maternity care during NVD and immediate postpartum care.

## CONCLUSIONS

The absence of informed consent and low levels of practices that ensure the universal rights of child-bearing women during midwifery care in the tertiary-level hospitals in Bangladesh are concerning. Inclusion of the RMC Charter endorsed universal rights of child-bearing women in standard protocols for clinical management in Bangladesh and their practice are essential to women’s right to dignity and respectful care in midwifery care.
