# Supplementary material for: Assessing informed consent practices during normal vaginal delivery and immediate postpartum care in tertiary-level hospitals of Bangladesh
Source: Eur J Midwifery. 2019 May 21;3:10. doi: 10.18332/ejm/109311 (PMC7839100; doi:10.18332/ejm/109311)
Supplement: Supplementary file 1 [file EJM-3-10-s1.pdf]

Supplementary Table 2: Questionnaire in English

Informed Consent Practices during Normal Vaginal Delivery and immediate postpartum care (within first 24 hours of delivery) in the tertiary level hospital setting, Dhaka, Bangladesh
Questionnaire for mothers

Code no: ....

Dear Respondent,
That all information is confidential and will be used for RESEARCH purposes only

What is the question about?

The question is to assess informed consent practices during normal vaginal delivery and immediate postpartum care (within first 24 hours of delivery) in the tertiary level hospital setting, Dhaka, Bangladesh.

NO	QUESTIONS	CODING CATEGORIES
**I.**	**Basic Information:**	
01	Date of Interview	______________________________ __
02	Place of Interview:	0. DMC<br>1. SSMC
03	Time of Interview:	______________________________ _
04	Time since Delivery:	______________________________ _
05	Delivery type	0. Spontaneous vaginal delivery<br>1.Assisted vaginal delivery
06	Place of Delivery	______________________________
II	**Mothers Socio-Demographic Characteristics**	
07	Woman's age in years:	______________________________ __
08	Mother Education	0. No education<br>1. Signature only<br>2. Primary education<br>3. Secondary and above
09	Mother employment status	0. Government<br>1. Private

NO	QUESTIONS		CODING CATEGORIES
			2. Household work
10	Monthly household income		0. Less than 5000TK<br>1. 5001-10000TK<br>2. Above 10000TK
11	Religion		0. Muslim 1. Hindu<br>2. Christian 3. Others
12	Living place		0. Rural 1. Urban 2. Peri urban
13	No. of total child birth including the current delivery (Total child birth= term +preterm + abortion + live births)		---
**III**	**Informed Consent Questions**		
14	Was consent taken by the service providers before proceeding NVD?		0. Yes 1. No<br>(If 'no' skip Q-15 & Q-16)
15.	What type of consent was taken?		0. Written 1. Verbal
16	Who gave the consent/assent?		0. Patient herself<br>1. Family members 2. Relatives
17	Did the service provider introduce him/herself before proceeding services?		0. Yes 1. No
18	Did the service providers allow you to ask any question regarding the services they provided?		0. Yes 1. No
19	Did the service providers explain and share the medication they provided to you?		0. Yes 1. No 2. N/A
20	After delivery, were you asked by service providers for consent before proceeding?	20.1 Abdominal exam	0. Yes 1. No 2. N/A
		20.2. Breast exam	0. Yes 1. No 2. N/A
		20.3. Any injection	0. Yes 1. No 2. N/A
		20.4. Anemia cyanosis	0. Yes 1. No 2. N/A
		20.5. Pulse measurement	0. Yes 1. No 2. N/A
		20.6. BP measurement	0. Yes 1. No 2. N/A
		20.7. Vaginal examination	0. Yes 1. No 2. N/A
		20.8. Blood transfusion	0. Yes 1. No 2. N/A
21	Which of the following issues of NVD explained during consent taking?		0. Purpose 1. Benefits<br>2. Success rate 3. Potential risks

NO	QUESTIONS	CODING CATEGORIES
22	If they asked for consent for a procedure, did they ask every time they do it?/ Did they ask for consent every time for the same procedure?	0. Yes 1. No 2. N/A
23	If consent is written, have they documented the signed consent in the patient record file?	0. Yes 1. No

Any other comments?

____

Interviewer's Name: __________________________ DATE _____/_____/______

Signature __________________________

© 2019 Karim M.A.
